# Endocarditis

**Published:** 1848-06

**Authors:** 


*Endocarditis.*—Depositions in the spleen almost always accompany endocarditis, and these correspond in character with the endocardial exudations, being purulent if the latter are purulent, and so on. Jaksch states, that the endocarditis following acute rheumatism, very frequently gives rise to pyæmia. That the formation of aneurism may sometimes be induced, when the texture of the heart has been weakened by carditis or endocarditis, appears evident from the details of a case, in which inflammatory exudation on the lining membrane of this organ co-existed with inflammatory softening of its whole structure at the apex, and in which, at this point, a considerable degree of attenuation had taken place, which might, no doubt, after some time, have gone on to rupture.—*Ibid.*

